# 2,4-Dihydroxy­benzaldehyde 4-methyl­thio­semicarbazone

**DOI:** 10.1107/S1600536808033308

**Published:** 2008-10-31

**Authors:** Kong Wai Tan, Chew Hee Ng, Mohd. Jamil Maah, Seik Weng Ng

**Affiliations:** a Department of Chemistry, University of Malaya, 50603 Kuala Lumpur, Malaysia; b Faculty of Engineering and Science, Universiti Tunku Abdul Rahman, 53300 Kuala Lumpur, Malaysia

## Abstract

The approximately planar mol­ecule of the title compound, C_9_H_11_N_3_O_2_S, is linked to adjacent mol­ecules by O—H⋯S hydrogen bonds to form a zigzag chain. Adjacent chains are consolidated by N—H⋯O hydrogen bonds into a two-dimensional array. An intramolecular O—H⋯N link is also present.

## Related literature

For the structure of isomeric 2,5-dihydroxy­benzaldehyde 4-methyl­thio­semicarbazone, see: Tan *et al.* (2008 ▶).
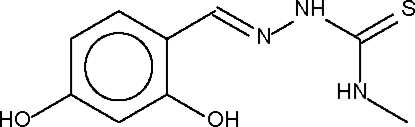


## Experimental

### 

#### Crystal data

C_9_H_11_N_3_O_2_S
*M* *_r_* = 225.27
Monoclinic, 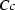

*a* = 18.0046 (6) Å
*b* = 4.6436 (1) Å
*c* = 12.2842 (4) Å
β = 106.695 (2)°
*V* = 983.74 (5) Å^3^
*Z* = 4
Mo *K*α radiation
μ = 0.31 mm^−1^
*T* = 100 (2) K
0.09 × 0.06 × 0.03 mm

#### Data collection

Bruker SMART APEX diffractometer
Absorption correction: multi-scan (*SADABS*; Sheldrick, 1996 ▶) *T*_min_ = 0.973, *T*_max_ = 0.991
4390 measured reflections
2128 independent reflections
1925 reflections with *I* > 2σ(*I*)
*R*_int_ = 0.034

#### Refinement

*R*[*F* ^2^ > 2σ(*F* ^2^)] = 0.038
*wR*(*F* ^2^) = 0.109
*S* = 1.11
2128 reflections
153 parameters
6 restraints
H atoms treated by a mixture of independent and constrained refinement
Δρ_max_ = 0.31 e Å^−3^
Δρ_min_ = −0.22 e Å^−3^
Absolute structure: Flack (1983 ▶), with 814 Friedel pairs
Flack parameter: 0.00 (1)

### 

Data collection: *APEX2* (Bruker, 2007 ▶); cell refinement: *SAINT* (Bruker, 2007 ▶); data reduction: *SAINT*; program(s) used to solve structure: *SHELXS97* (Sheldrick, 2008 ▶); program(s) used to refine structure: *SHELXL97* (Sheldrick, 2008 ▶); molecular graphics: *X-SEED* (Barbour, 2001 ▶); software used to prepare material for publication: *publCIF* (Westrip, 2008 ▶).

## Supplementary Material

Crystal structure: contains datablocks I, global. DOI: 10.1107/S1600536808033308/tk2316sup1.cif

Structure factors: contains datablocks I. DOI: 10.1107/S1600536808033308/tk2316Isup2.hkl

Additional supplementary materials:  crystallographic information; 3D view; checkCIF report

## Figures and Tables

**Table 1:** Hydrogen-bond geometry (Å, °)

*D*—H⋯*A*	*D*—H	H⋯*A*	*D*⋯*A*	*D*—H⋯*A*
O1—H1O⋯N1	0.84 (1)	1.93 (3)	2.694 (3)	151 (6)
O2—H2O⋯S1^i^	0.84 (1)	2.54 (1)	3.365 (2)	170 (4)
N2—H2N⋯O1^ii^	0.87 (1)	2.11 (1)	2.950 (4)	162 (3)

Symmetry codes: (i) 
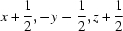
; (ii) 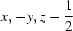.

**Fig. 1.**
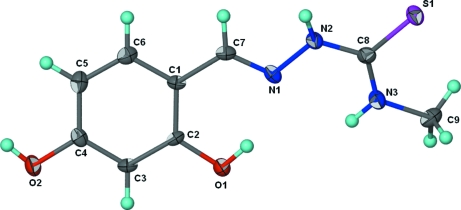
Thermal ellipsoid (Barbour, 2001) plot of (I) drawn at the 70% probability level. Hydrogen atoms are drawn as spheres of arbitrary radii.

## supplementary crystallographic information

### Comment

In continuation of on-going studies into the structural chemistry of thiosemicarbazones (Tan *et al.*,  2008), the title compound (I) was investigated. Molecule (I), Fig. 1, is essentially planar and is consolidated into a 2-D array by a combination of N-H···O and O-H···S hydrogen bonding contacts, Table 1.

### Experimental

4-Methylthiosemicarbazide (0.11 g, 1 mmol) and 2,4-dihydroxybenzaldehyde (0.14 g, 1 mmol) were heated in ethanol (10 ml) for 1 h. Slow evaporation of the solvent yielded yellow crystals.

### Refinement

Carbon-bound H-atoms were placed in calculated positions (C—H 0.95 to 0.98 Å) and were included in the refinement in the riding model approximation, with U_iso_>(H) set to 1.2-1.5 U_eq_(C). The hydroxy and amino H-atoms were located in a difference Fourier map, and were refined with distance retraints of O–H = 0.84±0.01 and N–H = 0.88±0.01 Å; their temperature factors were freely refined.

### Crystal data

**Table:**

C_9_H_11_N_3_O_2_S	*F*(000) = 472
*M**_r_* = 225.27	*D*_x_ = 1.521 Mg m^−^^3^
Monoclinic, *C**c*	Mo *K*α radiation, λ = 0.71073 Å
Hall symbol: C -2yc	Cell parameters from 1090 reflections
*a* = 18.0046 (6) Å	θ = 2.4–24.9°
*b* = 4.6436 (1) Å	µ = 0.31 mm^−^^1^
*c* = 12.2842 (4) Å	*T* = 100 K
β = 106.695 (2)°	Prims, yellow
*V* = 983.74 (5) Å^3^	0.09 × 0.06 × 0.03 mm
*Z* = 4	

### Data collection

**Table:**

Bruker SMART APEX diffractometer	2128 independent reflections
Radiation source: fine-focus sealed tube	1925 reflections with *I* > 2σ(*I*)
graphite	*R*_int_ = 0.034
ω scans	θ_max_ = 27.5°, θ_min_ = 2.4°
Absorption correction: multi-scan (SADABS; Sheldrick, 1996)	*h* = −23→22
*T*_min_ = 0.973, *T*_max_ = 0.991	*k* = −6→6
4390 measured reflections	*l* = −15→15

### Refinement

**Table:**

Refinement on *F*^2^	Secondary atom site location: difference Fourier map
Least-squares matrix: full	Hydrogen site location: inferred from neighbouring sites
*R*[*F*^2^ > 2σ(*F*^2^)] = 0.038	H atoms treated by a mixture of independent and constrained refinement
*wR*(*F*^2^) = 0.109	*w* = 1/[σ^2^(*F*_o_^2^) + (0.0598*P*)^2^] where *P* = (*F*_o_^2^ + 2*F*_c_^2^)/3
*S* = 1.11	(Δ/σ)_max_ = 0.001
2128 reflections	Δρ_max_ = 0.31 e Å^−^^3^
153 parameters	Δρ_min_ = −0.22 e Å^−^^3^
6 restraints	Absolute structure: Flack (1983), 814 Friedel pairs
Primary atom site location: structure-invariant direct methods	Flack parameter: 0.00 (1)

### Special details

**Table:**

Geometry. All e.s.d.'s (except the e.s.d. in the dihedral angle between two l.s. planes) are estimated using the full covariance matrix. The cell e.s.d.'s are taken into account individually in the estimation of e.s.d.'s in distances, angles and torsion angles; correlations between e.s.d.'s in cell parameters are only used when they are defined by crystal symmetry. An approximate (isotropic) treatment of cell e.s.d.'s is used for estimating e.s.d.'s involving l.s. planes.

### Fractional atomic coordinates and isotropic or equivalent isotropic displacement parameters (Å^2^)

**Table:**

	*x*	*y*	*z*	*U*_iso_*/*U*_eq_	
S1	0.50003 (5)	0.63599 (16)	0.50001 (6)	0.01776 (19)	
O1	0.65168 (12)	−0.1181 (5)	0.93136 (19)	0.0174 (5)	
O2	0.84529 (13)	−0.8242 (5)	1.05294 (19)	0.0198 (5)	
N1	0.63927 (15)	0.1424 (5)	0.7309 (2)	0.0144 (5)	
N2	0.60440 (15)	0.2962 (6)	0.6322 (2)	0.0147 (5)	
N3	0.53164 (15)	0.5567 (6)	0.7241 (2)	0.0166 (6)	
C1	0.73692 (17)	−0.2114 (7)	0.8141 (2)	0.0128 (6)	
C2	0.71210 (17)	−0.2660 (7)	0.9113 (2)	0.0122 (6)	
C3	0.74812 (18)	−0.4725 (7)	0.9891 (3)	0.0141 (6)	
H3	0.7300	−0.5117	1.0530	0.017*	
C4	0.81112 (16)	−0.6232 (6)	0.9739 (2)	0.0142 (6)	
C5	0.83798 (18)	−0.5674 (7)	0.8800 (3)	0.0174 (7)	
H5	0.8815	−0.6688	0.8704	0.021*	
C6	0.80096 (17)	−0.3645 (7)	0.8017 (3)	0.0159 (7)	
H6	0.8192	−0.3274	0.7378	0.019*	
C7	0.69685 (17)	−0.0131 (7)	0.7250 (2)	0.0142 (6)	
H7	0.7142	0.0020	0.6590	0.017*	
C8	0.54711 (17)	0.4887 (7)	0.6279 (3)	0.0149 (6)	
C9	0.47552 (19)	0.7743 (8)	0.7317 (3)	0.0206 (7)	
H9A	0.4879	0.8460	0.8099	0.031*	
H9B	0.4234	0.6899	0.7100	0.031*	
H9C	0.4773	0.9340	0.6804	0.031*	
H1O	0.633 (3)	−0.017 (11)	0.874 (3)	0.08 (2)*	
H2O	0.8796 (19)	−0.909 (8)	1.032 (4)	0.038 (13)*	
H2N	0.6097 (19)	0.218 (7)	0.5703 (17)	0.011 (8)*	
H3N	0.5552 (19)	0.476 (7)	0.7898 (17)	0.018 (9)*	

### Atomic displacement parameters (Å^2^)

**Table:**

	*U*^11^	*U*^22^	*U*^33^	*U*^12^	*U*^13^	*U*^23^
S1	0.0196 (4)	0.0180 (4)	0.0138 (3)	0.0027 (4)	0.0018 (3)	0.0021 (4)
O1	0.0193 (13)	0.0174 (12)	0.0159 (11)	0.0038 (9)	0.0059 (9)	0.0029 (10)
O2	0.0216 (12)	0.0196 (13)	0.0171 (12)	0.0069 (10)	0.0037 (9)	0.0040 (9)
N1	0.0159 (13)	0.0126 (13)	0.0125 (12)	−0.0018 (11)	0.0008 (10)	0.0013 (10)
N2	0.0176 (13)	0.0163 (13)	0.0097 (13)	0.0024 (11)	0.0030 (10)	−0.0001 (11)
N3	0.0195 (14)	0.0144 (13)	0.0152 (13)	0.0023 (11)	0.0037 (10)	0.0014 (10)
C1	0.0153 (14)	0.0135 (15)	0.0103 (14)	−0.0029 (12)	0.0048 (11)	−0.0005 (12)
C2	0.0105 (14)	0.0147 (15)	0.0129 (15)	−0.0017 (12)	0.0056 (11)	−0.0034 (12)
C3	0.0162 (15)	0.0159 (16)	0.0120 (15)	−0.0032 (12)	0.0069 (12)	−0.0012 (11)
C4	0.0148 (16)	0.0139 (14)	0.0113 (14)	−0.0007 (12)	−0.0006 (12)	0.0000 (12)
C5	0.0155 (16)	0.0163 (16)	0.0180 (17)	0.0012 (12)	0.0010 (12)	−0.0031 (12)
C6	0.0121 (15)	0.0214 (18)	0.0134 (15)	−0.0031 (14)	0.0023 (12)	−0.0033 (13)
C7	0.0157 (16)	0.0164 (16)	0.0117 (15)	−0.0035 (13)	0.0058 (12)	−0.0016 (12)
C8	0.0154 (15)	0.0127 (15)	0.0175 (16)	−0.0031 (12)	0.0064 (12)	0.0002 (12)
C9	0.0169 (16)	0.0266 (19)	0.0201 (17)	0.0003 (13)	0.0079 (13)	−0.0038 (13)

### Geometric parameters (Å, °)

**Table:**

S1—C8	1.699 (3)	C1—C2	1.413 (4)
O1—C2	1.367 (4)	C1—C7	1.454 (4)
O1—H1O	0.838 (10)	C2—C3	1.378 (4)
O2—C4	1.360 (4)	C3—C4	1.391 (4)
O2—H2O	0.836 (10)	C3—H3	0.9500
N1—C7	1.283 (4)	C4—C5	1.397 (4)
N1—N2	1.392 (3)	C5—C6	1.374 (4)
N2—C8	1.354 (4)	C5—H5	0.9500
N2—H2N	0.871 (10)	C6—H6	0.9500
N3—C8	1.328 (4)	C7—H7	0.9500
N3—C9	1.451 (4)	C9—H9A	0.9800
N3—H3N	0.880 (10)	C9—H9B	0.9800
C1—C6	1.400 (4)	C9—H9C	0.9800
			
C2—O1—H1O	106 (4)	C3—C4—C5	120.5 (3)
C4—O2—H2O	109 (3)	C6—C5—C4	119.5 (3)
C7—N1—N2	114.1 (3)	C6—C5—H5	120.3
C8—N2—N1	121.4 (3)	C4—C5—H5	120.3
C8—N2—H2N	121 (2)	C5—C6—C1	121.4 (3)
N1—N2—H2N	114 (2)	C5—C6—H6	119.3
C8—N3—C9	123.4 (3)	C1—C6—H6	119.3
C8—N3—H3N	123 (3)	N1—C7—C1	123.2 (3)
C9—N3—H3N	114 (3)	N1—C7—H7	118.4
C6—C1—C2	118.1 (3)	C1—C7—H7	118.4
C6—C1—C7	119.1 (3)	N3—C8—N2	118.3 (3)
C2—C1—C7	122.8 (3)	N3—C8—S1	123.4 (2)
O1—C2—C3	117.7 (3)	N2—C8—S1	118.3 (2)
O1—C2—C1	121.6 (3)	N3—C9—H9A	109.5
C3—C2—C1	120.8 (3)	N3—C9—H9B	109.5
C2—C3—C4	119.8 (3)	H9A—C9—H9B	109.5
C2—C3—H3	120.1	N3—C9—H9C	109.5
C4—C3—H3	120.1	H9A—C9—H9C	109.5
O2—C4—C3	117.9 (3)	H9B—C9—H9C	109.5
O2—C4—C5	121.6 (3)		
			
C7—N1—N2—C8	−174.4 (3)	C4—C5—C6—C1	0.2 (5)
C6—C1—C2—O1	177.7 (3)	C2—C1—C6—C5	1.4 (4)
C7—C1—C2—O1	−4.9 (4)	C7—C1—C6—C5	−176.0 (3)
C6—C1—C2—C3	−2.5 (4)	N2—N1—C7—C1	−174.0 (3)
C7—C1—C2—C3	174.8 (3)	C6—C1—C7—N1	−177.7 (3)
O1—C2—C3—C4	−178.3 (3)	C2—C1—C7—N1	5.0 (5)
C1—C2—C3—C4	1.9 (5)	C9—N3—C8—N2	175.6 (3)
C2—C3—C4—O2	179.7 (3)	C9—N3—C8—S1	−3.1 (4)
C2—C3—C4—C5	−0.2 (5)	N1—N2—C8—N3	8.8 (4)
O2—C4—C5—C6	179.3 (3)	N1—N2—C8—S1	−172.5 (2)
C3—C4—C5—C6	−0.9 (5)		

### Hydrogen-bond geometry (Å, °)

**Table:**

*D*—H···*A*	*D*—H	H···*A*	*D*···*A*	*D*—H···*A*
O1—H1O···N1	0.84 (1)	1.93 (3)	2.694 (3)	151 (6)
O2—H2O···S1^i^	0.84 (1)	2.54 (1)	3.365 (2)	170 (4)
N2—H2N···O1^ii^	0.87 (1)	2.11 (1)	2.950 (4)	162 (3)

Symmetry codes: (i) *x*+1/2, −*y*−1/2, *z*+1/2; (ii) *x*, −*y*, *z*−1/2.
